# 

**Published:** 1903-04

**Authors:** 


### CONTENTS.

ORIGINAL ARTICLES:
- Infectious Diseases of Our Farm Animals. By W. H. DALRYMPLE, M.R.C.V.S. . . . 201
- Gastro-Enterotomy, With Report of a Case. By J. MILLER, V.S. . . . 212
- Ulcerative Enteritis in the Horse. By G. L. BUFFINGTON, D.V.M. . . . 218
- Foot-and-Mouth Disease. By AUSTIN PETERS, M.R.C.V.S. . . . 222
- Vitality and Immunity. By CARL SCHULIN, M.D. . . . 233

ABSTRACTS FROM FOREIGN JOURNALS . . . 241

REPORTS OF CASES:

- Traumatic Pericarditis in a Cow. By JOHN J. REPP, V.M.D. . . . 247

- Peritoneal Filariasis in the Horse. By JOHN J. REPP, V.M.D. . . . 248

- Parturient Paralysis. By Dr. D. R. KOHLER . . . 24

EDITORIALS:

- Among the Colleges . . . 250

- Iowa's Action . . . 250

COMMENCEMENT EXERCISES . . . 251

NOTES . . . 256

SOCIETY PROCEEDINGS . . . 260

PERSONALS . . . 268

